# Molecular Characterization of Atypical Fibroxanthoma and Pleomorphic Dermal Sarcoma

**DOI:** 10.3390/cancers17111785

**Published:** 2025-05-27

**Authors:** Jason C. Klein, Breelyn A. Wilky, Heide L. Ford

**Affiliations:** 1 Department of Dermatology, Memorial Sloan Kettering Cancer Center, New York, NY 10065, USA; 2 Department of Dermatology, University of Colorado Anschutz Medical Campus, Aurora, CO 80045, USA; 3 Department of Medicine, University of Colorado Anschutz Medical Campus, Aurora, CO 80045, USA; 4 Department of Pharmacology, University of Colorado Anschutz Medical Campus, Aurora, CO 80045, USA

**Keywords:** atypical fibroxanthoma, pleomorphic dermal sarcoma, undifferentiated pleomorphic sarcoma, soft tissue sarcoma, genetics

## Abstract

Atypical fibroxanthoma (AFX) and pleomorphic dermal sarcoma (PDS) are dermal-based sarcomas that fall along a spectrum with different rates of local recurrence and metastasis. While AFX is less aggressive and confined to the dermis, PDS invades the subcutis. These tumors are most likely of mesenchymal origin, although they share common mutations with undifferentiated squamous cell carcinoma. Due to the rarity of these tumors, few studies have examined their molecular composition and gene expression. Initial studies, including exome and bulk RNA sequencing, targeted DNA sequencing of gene panels, DNA methylation, and copy number analyses, have identified recurrent UV-induced mutations in *TP53*, *NOTCH*, *CDKN2A*, and the *TERT* promoter. Recently, the first scRNA-seq dataset in AFX and PDS identified *COL6A3* as a novel biomarker. In this review, we synthesize the above datasets and discuss our current understanding of the molecular drivers and prognostic biomarkers in these tumors.

## Simple Summary

Atypical fibroxanthoma (AFX) and pleomorphic dermal sarcoma (PDS) are two soft tissue sarcomas that fall along a spectrum with different severities of patient outcomes. Several molecular studies have been performed in these tumors including mutation, expression, and epigenetic analyses. Here, we review molecular drivers and prognostic biomarkers in these tumors to highlight our current understanding of their biology. AFX and PDS are indistinguishable based on current molecular studies and diagnosis is based on depth of invasion at surgical removal. Both tumors are driven by UV-induced mutations in tumor suppressor genes and the *TERT* promoter. Recent scRNA-sequencing identified *COL6A3* as a potential biomarker in these tumors.

## 1. Introduction

Soft tissue sarcomas are a group of approximately 70 heterogeneous tumors [1,2]. Each individual subtype is rare, and as a group, they account for approximately 1% of adult cancers [3,4]. The most common subtypes of soft tissue sarcomas are liposarcoma (12%), leiomyosarcoma (12%), and undifferentiated pleomorphic sarcoma (UPS) (11%). UPS itself is a broad family of pleomorphic sarcomas, including tumors of internal organs, the retroperitoneum, and bones. Historically, this group also included dermal-based neoplasms.

Atypical fibroxanthoma (AFX) and pleomorphic dermal sarcoma (PDS) are dermal-based soft tissue sarcomas that most commonly occur on the head and neck of older male adults [5,6,7]. Both present as solitary, rapidly-growing ulcerated subcutaneous nodules [8,9]. On pathology, the differential includes spindled or sarcomatoid squamous cell carcinoma, leiomyosarcoma, and spindle cell melanoma, among others [10]. High-risk features associated with PDS include invasion into the subcutis, tumor necrosis, lymphovascular invasion, and perineural invasion [11].

PDS has a higher 5-year risk of local recurrence and metastasis compared to AFX (17% vs. 10% and 16% vs. 0.8%, respectively), and therefore, follow-up imaging is recommended [5]. In advanced cases of PDS, systemic treatments including doxorubicin, adriamycin, ifosfamide, and immune checkpoint inhibitors can be used [12,13,14]. Therefore, the distinction is important in providing accurate counseling on prognosis and surveillance. However, this diagnosis generally cannot be made with a superficial biopsy.

A better understanding of the molecular drivers of AFX and PDS is important in characterizing the etiology, behavior, and potential therapeutic targets for these tumors. In this review, we synthesize the current knowledge of the genetic drivers of AFX and PDS, including targeted gene sequencing, exome sequencing, DNA methylation, copy number variation, bulk RNA sequencing, and single-cell RNA sequencing (scRNA-seq) (Table 1).

## 2. Main Body

### 2.1. History of Atypical Fibroxanthoma and Pleomorphic Dermal Sarcoma

The entities of AFX and PDS have been described for over fifty years, although the nomenclature for these neoplasms has changed. Initially, these tumors fell under the umbrella of malignant fibrous histiocytoma before this term was reclassified as UPS in the World Health Organization classification in 2013 [23]. As UPS also includes tumors of deeper origin, some referred to dermal-based neoplasms as “UPS of the skin” [24,25]. In 2012, Christopher Fletcher coined the term “pleomorphic dermal sarcoma” (PDS) to define a dermal-based UPS that invades the subcutis [26]. This created a spectrum with atypical fibroxanthoma (AFX), which is confined to the dermis [27].

The first genetic study of AFX in 1994 focused on p53 because UV-related mutations in *TP53* had been described in other skin cancers [28]. Immunohistochemistry and single-strand conformation polymorphism analysis was performed to identify that 7/10 cases harbored *TP53* mutations. This finding was validated in 2001 by Sakamoto et al. in seven AFX and four superficial malignant fibrous histiocytoma cases (which would now be classified as PDS). *TP53* mutations were identified in four of the AFX cases and one PDS case [29]. Sakamoto et al. also looked for mutations in the RAS gene family but did not find any in the eight AFX cases studied [30]. Due to the role of *TERT* promoter mutations in melanoma and other cancers, Griewank et al. performed Sanger sequencing of the promoter in 25 AFX and 26 PDS tumors [31]. Mutations, most commonly UV-signature mutations, were identified in 93% of the AFX and 76% of the PDS tumors.

With the advent of next-generation sequencing and exome sequencing, groups have taken a more unbiased approach to identifying molecular drivers in AFX and PDS [32,33]. We will focus on those findings in the following sections.

### 2.2. DNA Sequencing of Targeted Gene Panels

Instead of examining a single target of interest as had been completed for *TP53*, *RAS*, and the *TERT* promoter above, targeted gene panels allow for high-depth coverage of a panel of known oncogenes and tumor suppressor genes [34]. Ak et al. performed the FoundationOne-CDx gene panel covering over 300 cancer genes on 10 AFX and 13 PDS patients [15]. Within the gene panel, they identified a high mutational burden of 54.59 mutations per megabase in the AFX samples and 69.49 mutations per megabase in the PDS samples. A total of 65 mutations with known and likely somatic impact were identified across the 23 tumors. Recurrent mutations were identified in *ASXL1* (3/23 tumors), *CD22* (8/23), *CDH1* (5/23), *CDKN2A* (17/23), *CUL4* (3/23), *DAXX* (3/23), *GATA4* (6/23), *KRAS* (2/23), *MPL* (3/23), *NOTCH1* (13/23), *NOTCH2* (6/23), *PIK3CA* (3/23), *TERT* promoter (15/23), and *TP53* (23/23).

Griewank et al. performed targeted sequencing of 341 oncogenes and tumor suppressor genes in 13 AFX tumors [16]. Overlapping genes identified by Ak et al. include *CDKN2A* (5/13 tumors), *NOTCH1* (5/13), *NOTCH2* (2/13), *TERT* promoter (12/13), and *TP53* (10/13). Griewank also identified mutations in *FAT1* (7/13) and *TSC2* (3/13).

Helbig et al. performed targeted sequencing of 17 hotspot genes in five AFX and five PDS tumors [17]. Recurrent mutations were only identified in *TP53* (5/5 PDS); however, this was a smaller analysis than the above studies.

*CDKN2A* and *TP53* were mutated in all three cohorts (23/46 and 38/46, respectively). *NOTCH1*, *NOTCH2*, and the *TERT* promoter were mutated in both the Ak et al. and Griewank et al. cohorts (18/36, 8/36, and 27/36, respectively).

### 2.3. Exome Sequencing

Only ~1–2% of the human genome encodes for proteins [35]. While the remainder of the genome contains regulatory sequences that control the spatiotemporal expression of genes, many studies focus on mutations directly altering protein function as these often have high penetrance [36]. Particularly when whole-genome sequencing is cost prohibitive, exome sequencing allows for the enrichment of protein-coding sequences [37].

Lai et al. performed exome sequencing on eight AFX tumors and patient-paired normal keratinocytes with a mean coverage of 113x. AFX had a very high mutational burden of 64 mutations per megabase. More than 70% of mutations were UV-signature mutations. A total of 49 genes were mutated in at least 75% of samples. Re-analysis of these data, including only nonsense, insertions, deletions, and splice variants, identified 10 genes mutated in at least two samples: *FAT1* (5/8), *TTN* (3/8), *PCLO* (2/8), *DNAH5* (2/8), *CUBN* (2/8), *SPTA1* (2/8), *XIRP2* (2/8), *DNAH11* (2/8), and *TDRD15* (2/8).

Klein et al. performed exome sequencing on 28 individuals with PDS [19]. Consistent with other studies, they identified a high mutational burden of 42.7 mutations per megabase. Recurrent nonsense, insertions, deletions, and splice variants were identified in five genes: *TP53* (11/28), *CDKN2A/B* (26/28), *DNHD1* (3/28), *PDGFRA* (4/28), and *KIT* (3/28).

Lim et al. performed exome sequencing on four AFX and two PDS tumors [20]; however, they did not provide a breakdown of mutations by class. Genes mutated in at least two tumors include *TP53* (6/6), *CSMD3* (5/6), *PKHD1L1* (5/6), *XIRP2* (5/6), *DNAH7* (5/6), *FBN1* (5/6), *HMCN1* (4/6), *LRP2* (4/6), *TRPM6* (4/6), *LRRK2* (4/6), *PRKCB* (4/6), *RIMS2* (2/6), and *FNBP1* (2/6).

*TP53* (17/42), *XIRP2* (7/42), *CSMD3* (6/42), and *PKHD1L1* (6/42) were all mutated in two of the three studies above.

### 2.4. DNA Methylation

Changes in gene expression drive cellular phenotypes and contribute to evolution and disease [38,39]. Mutations in DNA, either within coding sequences or regulatory sequences, are one mechanism of altering cellular phenotype. DNA methylation is another mechanism of modulating gene expression without directly altering the DNA sequence [40]. Methylation profiles can identify a unique variety of tumor types, including osteosarcoma and rhabdomyosarcoma [41,42,43]. Therefore, Koelsche et al. attempted to differentiate AFX from PDS using methylation profiles [22].

Koelsche et al. performed DNA methylation profiling on 17 AFX and 15 PDS tumors and compared them with several tumor types including other atypical spindle-cell neoplasms such as cutaneous squamous cell carcinoma (cSCC), leiomyosarcoma, and UPS. They used the Illumina Infinium 450k BeadChip and EPIC/850k BeadChip to measure DNA methylation. All samples were clustered, and t-SNE analysis was performed to delineate classes of DNA methylation. AFX and PDS samples were indistinguishable based on their methylation profiles but did separate from the remaining tumor types.

Basal cell carcinoma and cSCC showed the most similar methylation profiles to AFX and PDS. Interestingly, cSCC and basal cell carcinoma showed more similar profiles than UPS from deep soft tissue. However, AFX, PDS, basal cell carcinoma, and cSCC all came from the same institution, while the other tumors, clustering separately on the t-SNE, came from different centers. It is, therefore, possible that a difference in tissue handling or processing influenced the clustering of the tumors from different centers in this study.

### 2.5. Copy Number Variation

Genomic instability in cancer often leads to gains and losses of DNA, known as copy number variation [44]. Certain regions of the genome are repeatedly altered throughout many cancers, while others are unique to specific tumors [45]. These variations in copy number can involve oncogenes or tumor suppressors and lead to the progression of disease. Genomic hybridization is one method to quantify copy number changes.

Mihic-Probst et al. performed comparative genomic hybridization on 24 AFX samples [21]. They identified copy number alterations in 20 of the 24 AFX samples, with the most common being losses on chromosomes 9p (54%), which includes *CDKN2A*, and 13q (42%). Other losses included 3p, 10q, 11p (each 4%), 4q, 6q, 18q (each 8%), and Y (25%). Gains were identified in 4q, 5p, and 8q (each 4%).

Griewank et al. performed array-based comparative genomic hybridization on 20 AFX and 22 PDS samples [16]. There were no differences in patterns of copy number changes between the AFX and PDS samples. Both AFX and PDS shared losses of 8p, 9p, 9q, and larger deletions of chromosomes 13, 16, and 18. While losses were more common, there were gains in chromosomes 1q, 8q, 17q, and 19p.

Koelsche et al. performed copy number analysis based on the DNA methylation profiling of 17 AFX and 15 PDS tumors [22]. Similar to Griewank et al., they identified a greater number of copy number losses compared to gains. They identified losses of chromosomes 9p and 13q and gains in 8q. Additional gains were identified in individual samples including 5q, 8p, 13q, 11q, and 12q. Copy number alterations were similar in the cSCC samples studied, while the basal cell carcinomas showed fewer chromosomal gains and losses than AFX, PDS, and cSCC.

These data suggest that AFX and PDS are more prone to chromosomal losses than gains and that, in agreement with DNA sequencing, loss of *CDK2NA* is common in these tumors.

### 2.6. Bulk RNA Sequencing

Bulk RNA sequencing evaluates the relative expression level of genes and can aid in the diagnosis, prognosis, and personalized treatment of a variety of tumors [46]. Two groups have performed bulk RNA sequencing and differential gene analysis on dermal-based sarcomas.

Lai et al. performed RNA sequencing on eight matched AFX and normal samples and compared these to a publicly available fibroblast RNA-seq dataset and to The Cancer Genome Atlas [18]. They identified 8591 differentially expressed genes between AFX and unmatched keratinocytes and 4884 differentially expressed genes between AFX and nonmatched dermal fibroblasts. The overlapping 1446 differentially expressed genes were analyzed with gene set enrichment analysis. Defense response, immune system, and GPCR ligand binding were the most enriched pathways, while KRAS signaling was upregulated and p53 signaling was downregulated. Compared to the matched normal skin, AFX tumor samples demonstrated an upregulation of the tumor-associated macrophage (TAM) response (M2) and of epithelial–mesenchymal transition, which has been shown in other sarcomas [47,48,49].

Klein et al. performed RNA sequencing in 21 PDS samples and 6 cSCC samples [19]. Expression profiles of the PDS tumors clustered separately from the cSCC samples. Differential gene expression analysis revealed a significant enrichment of *PDGFRA/B* in PDS compared to cSCC. Moreover, they show that the expression profile of PDS is most similar to fibroblasts through a search using the All RNA-seq and ChIP-seq sample and signature search (ARCHS4).

### 2.7. Single-Cell RNA Sequencing

Recently, Klein et al. published single-cell RNA sequencing of three AFX and two PDS tumors [7]. The tumor cells clustered between fibroblasts and keratinocytes, suggesting a phenotype with characteristics from both cell types. The top differentially expressed gene in tumor cells compared to the other clusters was *CD74*, which has been proposed as a marker to distinguish AFX and PDS from other UPS [50,51]. In agreement with *COL3A1* overexpression from bulk RNA-seq of AFX versus fibroblasts and keratinocytes, they also found enrichment of collagen subunit genes in PDS tumor cells compared to other cell clusters in the sample (*COL4A1* and *COL4A2*) [18]. When comparing PDS to AFX samples, enriched genes were associated with cell matrix adhesion, blood vessel morphogenesis, and regulation of epithelial–mesenchymal transition. The top differential genes were also enriched for poor outcomes in other tumor types from the Human Protein Atlas. Both *COL6A1* and *BGN* were predictive of poor outcomes in UPS in two separate cohorts.

## 3. Conclusions and Future Directions

AFX and PDS share similar genetic backgrounds with no clear differential biomarkers on molecular analyses. These findings further support that AFX and PDS fall along a spectrum of the same tumor. Similar to cSCC, they are high mutational burden tumors (40–70 mutations per megabase) with a predominance of UV-signature mutations. The high mutational burden suggests that these tumors may be responsive to immune checkpoint inhibitors, which have been tested in several cases of advanced disease [14,52,53]. Frequently mutated genes are seen in other cancer types and include *TP53*, *TERT* promoter, *NOTCH1*, *CDKN2A*, and *FAT1*, all of which show mutations in at least 25% of the tumors analyzed (Table 2).

P53 is a key tumor suppressor and the most mutated gene in cancer [54]. It is mutated in almost 75% of the 149 tumors with targeted or exome sequencing reviewed in this manuscript.

Mutations in the *TERT* promoter can reactivate the expression of telomerase, allowing for continued cell replication in cancers [55]. Recurrent somatic mutations occur in about 29% of melanoma cases and 21% of cSCC cases [55,56]. While not captured in the exome sequencing cohorts, *TERT* promoter mutations were identified in 67% of AFX and PDS tumors with targeted gene sequencing. In 2024, the FDA approved Imetelstat, the first telomerase inhibitor, for myelodysplastic syndrome [57].

Notch signaling is a highly conserved pathway important in cell fate, organ development, and tissue homeostasis [58]. Dysregulation of Notch signaling in cancer promotes epithelial–mesenchymal transition and angiogenesis, leading to cancer proliferation, invasion, and metastasis. Both *NOTCH1* and *NOTCH2* were among the six most frequently mutated genes across all cohorts in this review. *NOTCH1* was mutated in 22% of tumors and *NOTCH2* was mutated in 10%.

*CDKN2A* encodes multiple tumor suppressor 1 (MTS1), which acts as an inhibitor to cyclin-dependent kinases 4 and 6 to prevent retinoblastoma protein phosphorylation [59]. Mutations in *CDKN2A* can remove this inhibition, allowing for retinoblastoma protein phosphorylation and abnormal cell cycle progression. Mutations, copy number variations, and methylation of *CDKN2A* have been associated with several cancer types. *CDKN2A* mutations were identified in 35% of AFX and PDS tumors included in this review with either targeted gene sequencing or exome sequencing. Moreover, chromosome 9p, which harbors *CDKN2A*, is the most common copy number variant in AFX and PDS, present in 34 of the 56 tumors (61%) included in this review.

*FAT1* is commonly mutated in human cancers (~20% in head and neck squamous cell carcinoma) and deletions accelerate tumor initiation and malignant progression and promote tumor stemness and spontaneous metastasis in cSCC models [60,61]. In AFX and PDS, we identified *FAT1* mutations in 51 of 126 tumors (40%).

**Figure 1:**
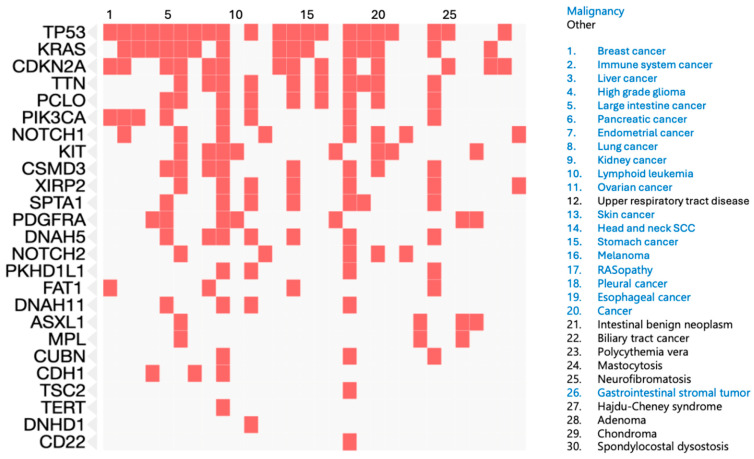
Diseases associated with frequently mutated genes in AFX and PDS. Blue text for disease indicates cancer.

Genes mutated in at least two AFX or PDS samples are enriched for roles in other cancers. The top ten diseases from the Jensen database enriched for the frequently mutated genes in AFX and PDS are all malignancies (breast cancer, immune system cancer, liver cancer, high-grade glioma, large intestine cancer, pancreatic cancer, endometrial cancer, lung cancer, kidney cancer, and lymphoid leukemia) (Figure 1). The top Reactome pathways enriched for these genes include PDGFR signaling, diseases of signal transduction by growth factor receptors and second messengers, KIT signaling, RUNX3 transcriptional regulation, and PI3K AKT signaling.

Both bulk and single-cell RNA sequencing have been performed on AFX and PDS tumors. Bulk RNA sequencing did not show any significant difference between AFX and PDS but, in concordance with the DNA analysis above, demonstrated downregulation of p53 signaling. It also demonstrated an immune signature, with an upregulation of the TAM response. TAMs promote cell invasion, intravasation, tumor stem cell viability, and induce angiogenesis [62]. They also suppress cytotoxic T and natural killer cells, preventing the ability of the immune system to attack the cancer. Single-cell RNA sequencing revealed enriched expression of *COL6A3* in a small cohort of PDS compared to AFX. In a different soft tissue sarcoma, undifferentiated pleomorphic sarcoma, *COL6A3* is predictive of poor outcomes [7]. Further work is ongoing to validate the mechanism and prognostic utility of *COL6A3* in soft tissue sarcomas.

Here, we provide an overview of our molecular understanding of AFX and PDS and provide insight into the genetic pathways that drive these tumors. While many of the studies discussed here are single-center studies with small sample sizes, many of the findings that we highlight have been replicated across studies. Further work is needed to clarify the relationship of AFX and PDS to cSCC, identify prognostic biomarkers for these tumors with varying patient outcomes, and identify novel therapeutic targets.

## Figures and Tables

**Table 1:** Key studies included in this review with the tumor type examined, assay used, and key findings.

Study	Tumors Examined	Assay Used	Key Findings
Ak et al. [15]	AFX and PDS	Targeted Gene Panel	Recurrent *TP53*, *TERT* promoter, *NOTCH1*, *CDKN2A* mutations
Griewank et al. [16]	AFX	Targeted Gene Panel and Copy Number Analysis	Recurrent *TP53*, *TERT* promoter, *NOTCH1*, *CDKN2A*, *FAT1* mutations. Recurrent losses of 8p, 9p, 9q, 13, 16, and 18.
Helbig et al. [17]	AFX and PDS	Targeted Gene Panel	Recurrent *TP53* mutations
Lai et al. [18]	AFX	Exome Sequencing and RNA sequencing	Recurrent *FAT1*, *XIRP2*, *TTN*, *CUBN*, *DNAH11*, *DNAH5*, *PCLO*, *SPTA1*, and *TDRD15*. KRAS signaling upregulated and p53 signaling downregulated.
Klein et al. [19]	PDS	Exome Sequencing and RNA sequencing	Recurrent *TP53*, *CDKN2A*, *PDGFRA*, *DNHD1*, and *KIT*. Expression profiling separates from cSCC, most similar to fibroblasts.
Lim et al. [20]	AFX and PDS	Exome Sequencing	Recurrent *TP53* mutations
Mihic-Probst et al. [21]	AFX	Copy Number Analysis	Recurrent losses of 9p and 13q
Koelsche et al. [22]	AFX and PDS	Copy Number Analysis and Methylation Profiling	Recurrent losses in 9p and 13q and gains in 8q. AFX and PDS indistinguishable from each other by methylation profiling.
Klein et al. [7]	AFX and PDS	Single-cell RNA sequencing	COL6A3 as a potential prognostic biomarker

**Table 2:** Genes identified in at least two different studies to be mutated in AFX or PDS. Total counts based on studies in which the gene was included. LOF: loss of function; GOF: gain of function.

Gene	Gene Function	Mutation Type	Study Design and Citation	Frequency
*TP53*	Tumor suppressor	LOF	Exome Sequencing [19,20] + Targeted Gene Panels [15,16,17]	108/149
*TERT* (promoter)	Telomere maintenance and cell immortalization	GOF	Targeted Gene Panels [15,16]	70/80
*NOTCH1*	Notch signaling	LOF	Targeted Gene Panels [15,16]	54/149
*CDKN2A*	Tumor suppressor	LOF	Exome Sequencing [19] + Targeted Gene Panels [15,16,17]	53/149
*FAT1*	Tumor suppressor	LOF	Exome Sequencing [18] + Targeted Gene Panels [16]	51/126
*NOTCH2*	Notch signaling	LOF	Targeted Gene Panels [15,16]	36/149
*XIRP2*	Actin filament organization	LOF	Exome Sequencing [18,20]	7/52
*CSMD3*	Associated with tumor mutational burden	LOF	Exome Sequencing [18,20]	6/52
*PKHD1L1*	Immune response	LOF	Exome Sequencing [18,20]	6/52
*PIK3CA*	Cell growth	GOF	Targeted Gene Panels [15,17]	4/149

## Data Availability

No new data were created for this review.

## Abbreviations

The following abbreviations are used in this manuscript:

AFX	Atypical fibroxanthoma
PDS	Pleomorphic dermal sarcoma
UPS	Undifferentiated pleomorphic sarcoma
cSCC	Cutaneous squamous cell carcinoma
scRNA-seq	Single-cell RNA sequencing
GOF	Gain of function
LOF	Loss of function
